# Canadian dietetic education and training actions to support Indigenization, decolonization, and reconciliation

**DOI:** 10.17269/s41997-025-01055-z

**Published:** 2025-06-12

**Authors:** Laura Correia Dias, Chelsea Leslie, Lee Rysdale, Victoria Emmell, Sandra A. Juutilainen, Shannan Grant, Kelly Gordon, Hannah Neufeld, Rhona M. Hanning

**Affiliations:** 1 School of Public Health Sciences, Faculty of Health, University of Waterloo, Waterloo, ON Canada; 2 NOSM University Dietetic Practicum Program, Thunder Bay, ON Canada; 3 NOSM University Dietetic Practicum Program, Sudbury, ON Canada; 4 School of Nutrition, Toronto Metropolitan University, Toronto, ON Canada; 5 Mount Saint Vincent University, Halifax, NS Canada; 6 Six Nations Health Services, Ohsweken, ON Canada

**Keywords:** Dietetics, Indigenous Peoples, Education, Canada, Diététique, Peuples autochtones, Éducation, Canada

## Abstract

**Objectives:**

The paper describes activities of dietetic education and training programs within Canada to advance Indigenization, decolonization, and reconciliation.

**Methods:**

A self-administered 34-question cross-sectional, online survey was distributed to all Canadian dietetic education and training programs, and available February–May 2022. A matrix question examined key actions and scope at program and/or institutional levels, while question logic and open-ended feedback options supported further description. Additional open-ended questions explored respondents’ perspectives on perceived barriers and needed supports for action. Descriptive statistics and deductive codes are presented.

**Results:**

The survey was completed by 20 of 29 dietetic programs (69%). Adding Indigenous-related content to curricula (*n* = 18) and cultural immersion opportunities within Indigenous settings (*n* = 16) mainly occurred at the program level. Attracting and supporting Indigenous students/interns, staff, and faculty and preceptors (*n* = 19) and providing cultural safety training to staff and faculty (*n* = 17) were common activities of institutions. Respondents identified lack of resources (funding, staff, and time) as systemic barriers and the need for dietetic leadership support to advance processes of Indigenization, decolonization, and reconciliation.

**Conclusion:**

This study established a baseline record of Indigenization, decolonization, and reconciliation activities in Canadian dietetic education and training programs that can inform future work. Programs are encouraged to (1) evaluate current and future activities; (2) ensure activities are part of a comprehensive approach to Indigenization, decolonization, and reconciliation, rooted in Indigenous, social justice, and health equity principles; and (3) consider collaborative action and advocacy to overcome systemic barriers, with the support of dietetic leadership.

**Supplementary Information:**

The online version contains supplementary material available at 10.17269/s41997-025-01055-z.

## Résumé

**Objectifs:**

Décrire les activités des programmes d’études et de formation en diététique au Canada qui font progresser l’autochtonisation, la décolonisation et la réconciliation.

**Méthode:**

Un sondage en ligne transversal auto-administré de 34 questions a été envoyé à tous les programmes d’études et de formation en diététique du Canada et diffusé entre février et mai 2022. Une question matricielle a porté sur les principales actions et leur portée à l’échelle du programme ou de l’établissement, tandis que la logique des questions et des options de rétroaction non dirigées ont favorisé des descriptions plus détaillées. D’autres questions ouvertes ont sondé l’opinion des répondants sur les obstacles perçus et les soutiens nécessaires à l’action. Les statistiques descriptives et les codes déductifs sont présentés.

**Résultats:**

Vingt programmes de diététique sur 29 (69%) ont répondu au sondage. L’ajout de contenus autochtones aux curriculums (n=18) et les possibilités d’immersion culturelle en milieu autochtone (n=16) existaient principalement à l’échelle des programmes. L’attraction et le soutien d’étudiants/de résidents, d’employés, de professeurs et de précepteurs autochtones (n=19) et la formation des employés et des professeurs à la sécurisation culturelle (n=17) étaient des activités courantes à l’échelle des établissements. Les répondants ont cerné le manque de ressources (financières, humaines et temporelles) comme étant des obstacles systémiques et le besoin de soutien de la part des leaders en diététique pour faire progresser l’autochtonisation, la décolonisation et la réconciliation.

**Conclusion:**

L’étude a constitué un dossier de référence des activités d’autochtonisation, de décolonisation et de réconciliation dans les programmes d’études et de formation canadiens en diététique pour éclairer les travaux futurs. Les programmes sont invités à: 1) évaluer leurs activités actuelles et futures; 2) s’assurer que ces activités s’inscrivent dans une démarche globale d’autochtonisation, de décolonisation et de réconciliation ancrée dans les principes autochtones, la justice sociale et l’équité en santé; et 3) envisager la concertation et le plaidoyer pour surmonter les obstacles systémiques, avec le soutien des leaders en diététique.

## Introduction

Indigenous Peoples of Turtle Island are resilient and are reclaiming the traditional knowledges and practices that supported their health and wellbeing pre-contact. Nevertheless, destructive impacts of colonization, including the Indian Residential School system of the 1880s through 1996, manifest in health inequities among Indigenous Peoples relative to non-Indigenous Peoples in what we now call Canada (Reading & Wien, 2009), including a lower life expectancy and higher prevalence of diet-related chronic diseases (Public Health Agency of Canada & Pan-Canadian Public Health Network, 2018). As health professionals, it is critical that dietitians be aware of traditional knowledges and practices about food, medicines, and health that support the self-determination and wellbeing of Indigenous Peoples, while also acknowledging the persistent negative historic, political, and socio-cultural impacts on health. Only then can we move forward in a way that supports culturally safe care (Greenwood et al., 2018).

In 2015, the Truth and Reconciliation Commission of Canada (TRCC) called for action to acknowledge the impacts of Indian residential schools, and to overhaul the systems perpetuating discrimination against Indigenous Peoples within Canada (Truth and Reconciliation Commission of Canada [TRCC], 2015). One of those systems listed in the TRCC’s 94 Calls to Action is healthcare, where leadership is called to, among other actions, intervene at the educational and training level to increase the number of Indigenous health professionals and the cultural competency of all healthcare professionals (TRCC, 2015). Despite having been published over 8 years ago, many of the 94 Calls to Action remain unachieved, including all seven actions related to health service provision (Jewell & Mosby, 2022). The existence of anti-Indigenous racism in contemporary healthcare (Turpel-Lafond, 2020) draws attention to the work that is still needed to address the Calls to Action, specifically Call #24. This Call requires healthcare providers to have skills-based training in intercultural competency, conflict resolution, human rights, and anti-racism (TRCC, 2015).

Progress towards addressing the healthcare-related Calls to Action has been slow. However, some action is being taken by healthcare professions across Canada, including the National Consortium for Indigenous Medical Education (2021) and the Canadian Association of Occupational Therapists (2019). Within dietetics, the most significant action came with the 2020 update of the Integrated Competencies for Dietetic Education and Practice (ICDEP). This comprehensive list of performance indicators set practice competencies for entry-level dietitians, and included five performance indicators focusing on Indigenous Peoples within Canada (Table 1) (Partnership for Dietetic Education & Practice (PDEP), 2020). These were in effect when PDEP ceased operations on October 31, 2024.

**Table 1:** Indigenous-focused performance indicators in the Canadian Integrated Competencies for Dietetic Education and Practice (Partnership for Dietetic Education & Practice, 2020)

**Food and nutrition expertise—Apply understanding of food environments**	
Performance Indicator 1.02 c	Demonstrate awareness of Indigenous values and ways of knowing related to food environments
**Food and nutrition expertise—Apply understanding of dietary practices**	
Performance Indicator 1.05 e	Demonstrate awareness of the role of Indigenous traditional/country foods in dietary practices
**Professionalism and ethics—Practice in a manner that promotes cultural safety**	
Performance Indicator 2.03 b	Demonstrate awareness of Indigenous values and ways of knowing related to health and wellness
Performance Indicator 2.03 c	Demonstrate an awareness of the ongoing impact of colonization, residential schools, intergenerational trauma, and systemic racism on Indigenous Peoples in Canada
**Management and leadership—Foster development of food skills in others**	
Performance Indicator 4.08 b	Demonstrate awareness of the availability and preparation of Indigenous traditional/country foods

To support the dietetics profession in developing a comprehensive approach to address the TRCC Calls to Action, the research project “Towards Decolonizing Canadian Dietetic Practice” (TDDP) was proposed to and funded by the Canadian Foundation for Dietetic Research in 2020. As part of the TDDP project, the main objective of the current study was to examine how Canadian dietetic education and training programs in 2022 were addressing Indigenization, decolonization, and reconciliation. The study also explored the perception of respondents on their dietetic programs’ barriers and needed support for action towards integrating the TRCC Calls to Action in a sustainable and meaningful way. This paper will share the results and interpret them in relation to ongoing actions to meet TRCC Calls to Action within the other healthcare professions in Canada, including identified challenges and opportunities. Establishing a baseline of current action within dietetic education and training is important to support the TDDP’s goal of developing a set of collaborative, action-oriented, and realistic recommendations for dietetic leadership to advance work on the TRCC Calls to Action.

## Methods

A cross-sectional survey was developed and pilot-tested in partnership with the Northern Ontario School of Medicine (NOSM) University Dietetic Practicum Program (DPP). The study received ethics clearance from the University of Waterloo Research Ethics Board (REB #42577). Participants’ consent was determined by affirmative responses to a series of questions before starting the survey, including consent for the use of anonymous data in publications.

### Survey design

The survey was developed based on existing literature on the integration of the TRCC Calls to Action/decolonization/Indigenization/cultural competence within education and standards of practice for healthcare professionals, as relevant to dietetics in Canada. The process of questionnaire development followed the guidelines of C.A. Woodward (Woodward, 1988) and was iterative, with feedback from the research team used to refine the survey questions and structure. The final survey comprised 34 questions that were primarily close ended (e.g., multiple choice). The survey was only available in English. Questions about consent and program information (Table 2) were mandatory, while all others were optional (Tables 3, 4, and 5). A matrix question was used to identify work towards Indigenization, decolonization, and reconciliation and the level at which it was occurring (program and/or institutional), for each key action area (Table 3). Recognizing that concepts can be interpreted differently, e.g., among Indigenous and non-Indigenous Peoples, participants were provided with the following definitions (Grafton & Melançon, 2020):

- Indigenization: “*Efforts to transform spaces, processes and institutions founded in non-Indigenous cultures to include Indigenous ways of knowing, being, and acting and the persons who practice them.*” (p. 149)
- Decolonization: Involves undoing colonial influences. For settlers, this means acknowledging and reversing internalized racist or discriminatory viewpoints; learning and drawing from Indigenous culture and knowledges; respecting and enabling the self-determination of Indigenous peoples. (p. 142)
- Reconciliation: “*Efforts to create mutually respectful relationships between Indigenous and non-Indigenous Peoples, or to benefit in terms of power and status from the appearance of such efforts.*” (p. 150)

Question logic was applied to guide respondents to provide details about activities they selected as “working on” in the matrix question. For most questions, open response boxes also invited participants to describe specific strategies used to address identified activities. Five open-ended questions were included at the end of the survey to explore respondents’ perspectives on barriers and needed support to advance Indigenization, decolonization, and reconciliation within dietetic education and training programs in Canada. While respondents were required to identify their dietetic education and/or training program type (undergraduate degree, post-undergraduate degree practicum, or combined masters practicum program), program size, primary respondent’s role, and identity as a Registered Dietitian or not, only anonymous data are presented. The survey was pilot tested by two Registered Dietitians working in the education setting. A complete copy of the revised survey, as administered, is available in Online Resource 1.

### Participants and survey administration

All accredited dietetic education and/or training programs in Canada were invited to participate in the survey (https://www.pdep.ca/accreditation/accredited-program-list.aspx). The survey was delivered using Qualtrics (Seattle, WA), an online survey tool, to facilitate reach across a large geographic area and provide flexibility for participants to respond when convenient. The survey link and information letter were emailed to those with leadership roles within these programs with instructions that the survey be completed by the individual or group with the best knowledge of current activities taking place in their program and/or institution aimed at advancing Indigenization, decolonization, and reconciliation. Responses were limited to one per program. The survey was available online from February to May 2022, and periodic emails were sent to programs to encourage response. No incentives for participation were provided.

### Data analysis

Data were imported from Qualtrics into Microsoft Excel (365, 2017, Redmond, WA) and organized into distinct sections to facilitate analysis, based on the survey’s sections and questions (i.e., matrix question, key action areas, open-ended questions). Frequency distribution was used to describe and summarize quantitative data (Eyler, 2021). The number of cases within selected categories (N) and the percentage of cases within the total sample (%) are described in Tables 2, 3, and 4. No further quantitative analysis was conducted.

Qualitative data from open response boxes in six sections of the survey (A–F, Table 4) were extracted. Given that the open-ended responses were brief and not conducive to a full-scale thematic analysis, relevant data (e.g., data relating to ongoing actions versus intentions for future action) were manually organized by topic and sub-topic (by LD) using a process akin to unstructured coding (Miles et al., 2018). The results were reviewed (by RMH) and are presented in Online Resource 2, with excerpts in the manuscript Results section. In addition to these brief comments on section-specific responses, the survey asked five open-ended questions about Indigenization, decolonization, and reconciliation (I/D/R):

1. Is there anything that your program/institution is doing on I/D/R that you would like to add?
2. Aside from your current progress towards I/D/R, what might you like to see your program/institution doing in the near future on this topic?
3. What are the three main systemic barriers you see for your program/institution to progress when it comes to I/D/R?
4. How might dietetics leadership in Canada (Dietitians of Canada, regulatory bodies, Partnership for Dietetic Education and Practice) help you advance I/D/R in your program/institution?
5. Do you have any final thoughts to share?

The questions became the deductive coding framework (Braun et al., 2019). Two investigators (LD, RMH) independently reviewed the data. The questions were distilled into three main themes (desired future actions, main systemic barriers, support needed from dietetics leadership to advance I/D/R). The text associated with each main theme was systematically coded to identify and organize key points considering frequency of mentions and indications of the importance (Creswell & Creswell, 2022). The reviewers met and established consensus on content for each theme; together they selected relevant quotations (Table 5).

## Results

### Respondents’ characteristics

The survey was completed by 20 of 29 dietetic education and training programs (69% response rate), with half being undergraduate degrees (*n* = 10) and more than half located in Central Canada (*n* = 12). In response to the request that the survey be completed by individuals or groups most familiar with activities related to Indigenization, decolonization, and reconciliation, most respondents held the title of program director/manager (*n* = 13), and 16 were Registered Dietitians. Program size ranged from 37 to 150 students for undergraduate degrees, 4 to 40 for post-undergraduate degree practicums, and 15 to 34 for combined masters practicum programs. Only one program indicated assessing candidates’ prior learning related to Indigenization, decolonization, and reconciliation; the program reported that fewer than 20% of candidates admitted had prior learning in this area. A summary of the respondents’ characteristics is available in Table 2.

**Table 2:** Respondents’ characteristics (*n* = 20/29 programs)

Characteristic	Option	*N*	%
**Program type**	Undergraduate degree	10	50
	⋅ Partially integrated (academic education with limited practicum training enrollment)^a^	3	15
	⋅ Non-integrated (academic education only)	4	20
	⋅ Fully integrated (academic education and practicum training)	3	15
	Post-undergraduate degree practicum	5	25
	Combined masters practicum program	5	25
**Location**	West Coast & Prairie Provinces	3	15
	Central Canada	12	60
	Atlantic Provinces	5	25
**Primary respondent’s role**	Program director/manager	13	65
	Internship coordinator	4	20
	Regional coordinator	0	0
	Professor/instructor	6	30
	Other. Please elaborate^b^	5	25
**Respondent is a Registered Dietitian**	Yes	16	80
	No	1	5
	Other. Please elaborate^c^	3	15

Program type as defined by the Partnership for Dietetic Education and Practice (https://www.pdep.ca/accreditation/accredited-program-list.aspx)

Geographic location informed by the Government of Canada (https://www.canada.ca/en/immigration-refugees-citizenship/corporate/publications-manuals/discover-canada/read-online/canadas-regions.html). West Coast & Prairie Provinces include the provinces of BC, AB, SK, and MB. Central Canada includes the provinces of ON and QC. The Atlantic provinces include the provinces of NB, NS, PEI, and NL. There are no dietetic programs in the territories

^a^Partially integrated undergraduate degrees: only a percentage of undergraduate students enrolled in the undergraduate degree are provided access the practicum training portion of the program

^b^Other responses added included curriculum chair, associate department head, science curriculum review, internship preceptor, dietetic educator

^c^Other responses included MSc and PhD (dietetics-related degrees), more than one person was involved in responding to the survey, but only one is a RD (two respondents)

### Indigenization, decolonization, and reconciliation action at the program level and/or institutional level

Table 3 describes areas of action by program and institution level (and areas of non-action) under education and training, professional practice, and strategic planning, policies, and quality improvement. Most respondents reported currently incorporating content on Indigenous Peoples in the curriculum at program (90%) and institutional levels (75%). Most programs also offered cultural immersion placements and activities within Indigenous settings, such as communities or organizations (80%). Respondents also indicated activities towards providing cultural safety and/or anti-racism training for staff/faculty (85%), having an Indigenous advisory council/board (100%), and developing policies focused on Indigenization, decolonization, and reconciliation (82%), though these activities were more often at the institutional level. Most institutions were also reported to have a strategic plan focused on Indigenization, decolonization, and reconciliation (85%) and to be working towards attracting and supporting Indigenous Peoples (students/interns, staff and faculty, preceptors) (95%) and establishing partnerships with Indigenous Peoples (85%).

**Table 3:** Areas of Indigenization, decolonization, and reconciliation action at the dietetic program level, institutional level, or both (*n* = 20/29)

	Yes, at the program level		Yes, at the institutional level		No	
Area of action	*N*	%	*N*	%	*N*	%
**Education and training**						
Attracting and supporting Indigenous Peoples (students/interns, staff and faculty, preceptors)	13	65	19	95	0	0
Curriculum content on Indigenous Peoples	18	90	15	75	1	5
Cultural immersion practices (i.e., practicum placements in Indigenous communities)^a^	16	80	7	35	3	15
**Professional practice**						
Cultural safety and/or anti-racism training^b^ for staff and faculty	10	50	17	85	2	10
Partnership with Indigenous Peoples (i.e., access to Elders/Knowledge Keepers)	11	55	17	85	1	5
**Strategic planning, policies, quality improvement**						
Strategic planning	13	65	17	85	0	0
Indigenous advisory council/board^c^	5	28	18	100	2	11
Policies regarding Indigenization, decolonization, and reconciliation^c^	1	6	14	82	6	35
Quality improvement (i.e., evaluation of current practices)	12	60	13	65	3	15

^a^Cultural immersion practices were defined for survey participants as also known as intentional placements, activities where students/interns can immerse in the culture of Indigenous Peoples in Canada. They include practicum placements or week-long camps

^b^Cultural safety and/or anti-racism training, as identified in the survey, could be delivered in-person or online, and include university-wide professional development courses, webinars (internal/external), and courses/workshops administered by external organizations. However, for the purpose of this survey, they had to include a focus on Indigenous Peoples in Canada

^c^*n* = 18

### Areas of action towards Indigenization, decolonization, and reconciliation

For those dietetic education and training programs that responded that they were taking action (Table 3), the details of the work being completed are outlined in Table 4 under the following sections: (A) Attracting and supporting Indigenous Peoples, (B) Curriculum content, (C) Cultural immersion practices, (D) Cultural safety/anti-racism training, (E) Partnerships with Indigenous Peoples, and (F) Strategic planning, policies, quality improvement.

**Table 4:** Details about work being completed by those dietetic education and training programs/institutions within Canada that indicated taking action towards Indigenization, decolonization, and reconciliation

Question	Response	*N*	%
**Section A—Attracting and supporting Indigenous Peoples**			
Approaches to attract and support Indigenous students/interns in the program/institution (*n* = 20 respondents)^a^	Yes	14	70
	No	3	15
Approaches in place (*n* = 15)	Prioritizing spots for Indigenous students/interns	9	60
	Financial support	8	53
	Improving access to Indigenous mentors	12	80
	Offering living/meeting spaces and events for Indigenous students/interns	7	47
	Dedicated academic support	6	40
	Supporting access to personal or cultural supports	9	60
	Increased accessibility	4	27
	Community outreach to Indigenous high school students	9	60
Approaches to attract and support Indigenous staff and faculty in the program/institution (*n* = 19)^a^	Yes	9	47
	No	5	26
Approaches in place (*n* = 13)	Prioritizing spots for Indigenous staff and faculty	10	77
	Financial incentives	1	8
	Dedicated spaces and events for Indigenous staff	4	31
	Professional development support	4	31
	Supporting access to personal or cultural supports	5	38
Approaches to support student access to Indigenous preceptors in the program/institution (*n* = 19)^a^	Yes. Please briefly elaborate on the types of approaches	9	47
	No	5	26
**Section B—Curriculum content on Indigenous Peoples**			
How the content on Indigenous Peoples in Canada is incorporated in the curriculum (*n* = 19)	Undergraduate, graduate, or internship course(s) or course content on Indigenous Peoples	16	84
	Guest lectures by Indigenous Peoples	14	74
	Field trips	1	5
	One-time events	11	58
	Course work	15	79
	Practicum placements	13	68
	Land-based learning	2	11
Designation of the courses on Indigenous Peoples in Canada (or with Indigenous content) (*n* = 16)^a^	Mandatory	5	31
	Elective	3	19
Topics usually covered in the courses/course content on Indigenous Peoples in Canada (*n* = 16)	Indigenous values and ways of knowing related to health and wellness	15	94
	Historic and ongoing impacts of colonization, Indian residential schools, intergenerational trauma, systemic racism	13	81
	Past and ongoing nutrition inequities	16	100
	Social determinants of health	16	100
**Section C—Cultural immersion practices**			
Availability of cultural immersion practices for all students/interns (*n* = 14)^a^	Yes, and they are required for all students/interns	0	0
	Yes, but they are not required for all students/interns	3	21
	No, there is no capacity to provide for all students	6	43
Type of cultural immersion practices offered to students/interns (*n* = 13)	Practicum placements/rotations in Indigenous communities/settings serving Indigenous Peoples	12	92
	One-time immersion opportunities	1	8
Availability of preparation to students/interns, in advance of cultural immersion opportunities (*n* = 13)^a^	Yes. Please describe:	8	62
	No	4	31
Availability of self-reflection/debriefing to students/interns, after a cultural immersion opportunity (*n* = 12)^a^	Yes. Please describe:	10	83
	No	0	0
**Section D—Cultural safety and/or anti-racism training for staff and faculty**			
Type of cultural safety and/or anti-racism training provided to staff and faculty (*n* = 17)	Webinars	13	76
	Courses	4	24
	Workshops	6	35
Is cultural safety and/or anti-racism training mandatory for staff and faculty? (*n* = 17)^a^	Yes, as part of the orientation process	0	0
	Yes, as part of ongoing human resources requirements	2	12
	No	12	71
**Section E—Partnership with Indigenous Peoples**			
Types of external partnerships with Indigenous Peoples communities and/or organizations (*n* = 15)	For cultural immersion practices	4	27
	For curriculum enhancement	6	40
	For strategic planning	8	53
	For supportive environments for Indigenous students/interns, staff and faculty, and preceptors	9	60
	For ongoing support with program	7	47
**Section F—Strategic planning, policies, quality improvement**			
** Strategic planning**			
Existence of a strategic plan that includes a focus on Indigenization, decolonization, and/or reconciliation (*n* = 17)^a^	Yes, there is a strategic plan for the program only	0	0
	Yes, there is a strategic plan for the institution only	4	24
	Yes, there is a strategic plan for the program and the institution	5	29
	No, there is no strategic plan for the program nor the institution with this focus	0	0
	No, but it is being developed for our program only	0	0
	No, but it is being developed for our institution only	1	6
	No, but it is being developed for our program and institution	3	18
** Indigenous advisory board/council**			
Existence of an Indigenous advisory council, board, or group (*n* = 18)^a^	Yes, only for our program	0	0
	Yes, only for the institution	8	44
	Yes, and it is shared between the program and the institution	0	0
	Yes, one for the program and a different one for the institution	3	17
	No, and no plans to have one in the future	0	0
	No, but planning is underway to have one in the future	1	6
** Policies regarding Indigenization, decolonization, and reconciliation**			
Focus of the policies regarding Indigenization, decolonization, and reconciliation (*n* = 12)	Attracting and supporting Indigenous Peoples	7	58
	Identifying and eradicating racism against Indigenous students and staff	6	50
	Establishing and maintaining relationships with Indigenous partners	7	58
	Conducting research with Indigenous Peoples	7	58
	Providing of tobacco, gifts, or honorariums to Indigenous Peoples	1	8
** Quality improvement**			
Is Indigenization, decolonization, and reconciliation action evaluated? (*n* = 17)^a^	Yes	5	29
	No	4	24
Actions that are subject to evaluation/monitoring (*n* = 11)	Attract and support Indigenous students/interns, staff and faculty, and preceptors	3	27
	Curriculum content on Indigenous Peoples in Canada	8	73
	Cultural immersion practices	1	9
	Cultural safety and/or anti-racism training for staff and faculty	4	36
	Partnerships with Indigenous Peoples	1	9
	Strategic planning	3	27
	Indigenous advisory groups	2	18
	Policies regarding Indigenization, decolonization, and reconciliation	3	27
	Incident reporting	3	27

^a^Where “*Yes*” and “*No*” responses do not add to the number of respondents per question, this was due to “*Other. Please elaborate*” or “*Not sure. Please elaborate*” responses, included in qualitative feedback (Table 5 and Online Resource 2)

#### Attracting and supporting Indigenous Peoples

Fourteen respondents reported having clear approaches in place within their programs to attract and support Indigenous students/interns. The most frequently adopted approach identified was improving access to Indigenous mentors (*n* = 12), often Indigenous faculty members within the program and/or institution (Online Resource 2). Another common approach identified was prioritizing spots for Indigenous students/interns in dietetic education and training programs (*n* = 9). Open-ended feedback revealed that this approach included automatically accepting Indigenous applicants for interviews or reserving a percentage of the available program spots for Indigenous applicants who meet the admissions requirements. Other admission-stage activities reported included using a scoring system favouring applicants who self-identify as Indigenous and a blinded admissions process (Online Resource 2). About half of respondents identified that approaches were also in place for attracting and supporting Indigenous staff/faculty. The main approach adopted was prioritizing spots for Indigenous staff/faculty in the program/institution (*n* = 10). For faculty positions, this often meant implementing cluster hiring initiatives focused on Indigenous candidates. Similarly, about half of respondents (*n* = 9) indicated supporting their students/interns’ access to Indigenous preceptors, often through collaboration with Indigenous dietitians working in Indigenous communities and/or collaborations with local Indigenous communities/organizations.

#### Curriculum content

Sixteen respondents identified that courses were available to their students/interns with content on Indigenous Peoples as the sole or partial focus. Overall, the Indigenous-related content is often scaffolded throughout the program curriculum (Online Resource 2). Many respondents (*n* = 15) incorporate content on Indigenous Peoples within course work, such as case studies, reflections, or readings. Open-ended feedback indicated that some respondents also rely on external Indigenous-focused resources (Online Resource 2). When courses focused on Indigenous content were available, only five respondents reported them as mandatory to attend. Of topics required by ICDEP, “*past & ongoing nutrition inequities and social determinants of health*” is covered by the 16 programs that responded to this question, while 15 programs also cover “*Indigenous values and ways of knowing related to health and wellness*” (Partnership for Dietetic Education & Practice, 2020). Some respondents added Indigenous food sovereignty and Indigenous agriculture as topics in their curriculum.

#### Cultural immersion practices

Of the 16 programs that reported providing Indigenous cultural immersion opportunities for students/interns, in no case were these mandatory. Indeed, six of the programs identified lacking capacity to make Indigenous cultural immersion opportunities available to all students/interns. As one respondent commented:

“*More immersive experiences for students would be great.*”

Cultural immersion opportunities often take the shape of placements of short (2 to 3 weeks) or long duration (4 to 8 weeks) within Indigenous settings (e.g., Aboriginal Health Access Centres) (*n* = 12), (Online Resource 2). Of those offering placements within Indigenous settings, many indicated preparing students/interns beforehand (*n* = 8), as well as debriefing upon completion (*n* = 10). Reportedly, such preparation and/or debrief is regular practice for program placements and not exclusive for those within Indigenous settings, although one program identified Indigenous cultural safety training before placements in Indigenous communities or settings.

#### Cultural safety/anti-racism training

Few respondents (*n* = 2) identified that their workplace makes it mandatory for staff/faculty to attend cultural safety and/or anti-racism training. Training was offered through webinars (*n* = 13), courses, or workshops by external institutions specialized in equity, diversity, and inclusion (*n* = 8); staff-maintained or institutional websites for resource-sharing; and journal clubs (Online Resource 2). One respondent noted wanting:

“*More workshops and training to learn and understand.*”

#### Partnerships with Indigenous Peoples

Of the 15 responding programs that identified having external partnerships with Indigenous Peoples, communities, and/or organizations, this was often to support Indigenous students/interns, staff, faculty, and preceptors (*n* = 9) or for strategic planning purposes (*n* = 8) (Table 4).

#### Strategic planning, policies, quality improvement

An institutional level–only strategic plan with a focus on Indigenization, decolonization, and reconciliation was reported by four respondents, while five have such planning at both program and institutional levels. Eight respondents have an Indigenous advisory council/board at the institutional level only, while three respondents have different advisory councils/boards for their program and for their institution. Indigenous advisory councils can also exist within partner institutions (e.g., hospitals hosting interns), be replaced by existing partnerships with Indigenous Peoples or facilitated by Indigenous staff (e.g., an Indigenous Initiatives Coordinator) (Online Resource 2). Most policies on Indigenization, decolonization, and reconciliation were reported to focus on attracting and supporting Indigenous Peoples (*n* = 8), establishing and maintaining relationships with Indigenous partners (*n* = 7), or conducting research in partnership with Indigenous Peoples (*n* = 7), such as requiring active engagement of Indigenous Peoples on ethics review. When asked whether activities towards Indigenization, decolonization, and reconciliation were evaluated, only five respondents gave a clear “yes.” However, curriculum content relevant to Indigenous Peoples was often evaluated as part of routine course evaluation and open-ended feedback indicated other evaluation activities, e.g., as part of program accreditation. One respondent reported receipt of a teaching and learning grant to evaluate the impact of exposing students to Indigenous leaders on Indigenous food sovereignty.

Open-ended feedback related to questions in Table 4 is available in Online Resource 2.

### Systemic barriers and supports needed

Open-ended feedback included elaboration on Indigenization, decolonization, and reconciliation action and responses to questions on desired next steps for the program; systemic barriers to progress; and identification of supportive action from dietetic leadership, including professional organizations and regulation and accreditation bodies (Table 5).

**Table 5:** Main responses to open-ended questions about desired next steps, barriers, and needed supports to advance Indigenization, decolonization, and reconciliation action for dietetic programs within Canada

Responses	Exemplar quotations
(1) **Aside from your current progress towards Indigenization, decolonization, and reconciliation, what might you like to see your program/institution doing in the near future, on this topic?**	
• More action on the strategies described in the survey • Complete a program-level strategic plan • Integrate Indigenous Peoples content in the overall program curriculum (not just individual courses) • More cultural immersion opportunities for students/interns • More practice-based research projects in collaboration with Indigenous Peoples • Understand potential barriers for Indigenous candidates to access dietetic programs • More cultural safety and/or anti-racism training for staff and faculty	“*Finalize strategic planning at the program level; integration of these issues into the curriculum at various levels rather than being “taught” in a course.*” “*I’d like to see action on all of the items noted throughout this survey. Some of the items have received attention, however much more needs to be done*.”
(2) **What are the three main systemic barriers you see for your program/institution to progress when it comes to Indigenization, decolonization, and reconciliation?**	
• Lack of resources: ◦ Lack of funding (i.e., to attract and support Indigenous students/interns) ◦ Lack of staff and/or faculty, including a lack of Indigenous faculty, such as Indigenous preceptors ◦ Lack of time (i.e., to learn and implement changes) ◦ Lack of indicators to conduct quality assurance on action towards Indigenization, decolonization, and reconciliation • Lack of knowledge (i.e., needed changes, negative impact of current policies) • Lack of support and commitment to change: ◦ Due to the lack of cultural and religious diversity within the institution and its surrounding community ◦ Due to lack of cultural and gender diversity within the institution’s highest administrative positions, such as dean or directors, and lack of representation of Indigenous Peoples in such positions • Competing priorities (can lead to less support to action towards Indigenization, decolonization, and reconciliation and/or less ability to fit Indigenous content within the program curriculum) • Size of the institution (too small to accommodate and prioritize needed changes; too big and it leads to inertia, and change takes longer to happen) • Inability to mandate ongoing cultural safety and anti-racism training to the program’s many external partners (i.e., preceptors) • Racism	“*I think the biggest systemic factor we face is lack of knowledge around what types of curricular changes would make a difference in terms of Indigenization, decolonization, and reconciliation. We have very few Indigenous faculty members and, as a result, we lack the expertise to advance this work*.” “*Having enough people to do the work, getting input from communities, Elders, being able to offer appropriate compensation are all challenges.*” “*Inertia—the University is a large institution and it can take a lot of time to make changes—one seemingly small change can impact layers of policy/systems*.”
(3) **How might dietetics leadership in Canada (Dietitians of Canada, regulatory bodies, Partnership for Dietetic Education and Practice) help you advance Indigenization, decolonization, and reconciliation in your program/institution?**	
• Offer resources: ◦ Offer new resources to support professional development ◦ Continue to offer existing resources (i.e., webinars) ◦ Support a community of practice/network ◦ Offer guidance (i.e., define Indigenization, decolonization, and reconciliation within dietetic education and practice, how to integrate these concepts in the curriculum, create indicators to measure progress and success) • Incorporate Indigenization, decolonization, and reconciliation in the provincial regulatory bodies’ professional practice requirements (i.e., annual renewal process, quality assurance program) • Lead by example (i.e., diversify their membership) • Require non-Canadian professionals seeking to enter dietetic practice in Canada to complete training focused on Indigenous Peoples (as done in New Zealand) • Incorporate actions to support Indigenization, decolonization, and reconciliation in the dietetic programs’ accreditation process • Advocate for necessary resources to support Indigenization, decolonization, and reconciliation initiatives in dietetic education and practice	“*Bringing educators together to share ideas and experiences would be wonderful*.” “*Continue to provide opportunities for learning (webinars, readings, Learning on Demand), *etc*.*” “*I would like to see this included in our professional practice requirements from our provincial regulating bodies*.”

Respondents identified wanting increased program/institutional action across all areas within the survey. One respondent offered the following suggestion for future actions:

“*Practice-based research project collaborations with Indigenous agencies, communities, RDs* [Registered Dietitians]*, and Peoples. Determining any program access barriers for Indigenous learners. Outreach to schools to attract more Indigenous learners to the profession and program.*”

Respondents indicated lack of resources as a key systemic barrier to advance Indigenization, decolonization, and reconciliation. More specifically, they expressed that action had been hindered by lack of funding, staff/faculty resources (especially Indigenous faculty), and time. Lack of knowledge on Indigenization, decolonization, and reconciliation; lack of support and commitment to change from program and/or institutional administration; and competing priorities at the program level were also systemic barriers identified by respondents. One respondent conveyed a challenge of advancing Indigenization, decolonization, and reconciliation:

“*Most of the people on my team are very keen to make changes, but finding the time to dedicate to that is always challenging, especially during the pandemic.*”

Regarding support, respondents frequently reported a need for resources. More specifically, respondents mentioned desiring professional development resources, a community of practice, and guidance on how to lead and implement Indigenization, decolonization, and reconciliation within Canadian dietetics. One respondent provided feedback on a potential focus of such guidance, to be provided by dietetic leadership in Canada (i.e., Dietitians of Canada (DC), regulatory bodies):

“*Define the scope of Indigenization, decolonization, and reconciliation in dietetic education and practice and how this is operationalized at the academic and practicum level*.”

Respondents also indicated that Indigenization, decolonization, and reconciliation considerations should be incorporated in the provincial regulatory bodies’ professional practice requirements (e.g., College of Dietitians annual membership renewal).

## Discussion

The discussion is organized according to the Results section: respondents’ characteristics; Indigenization, decolonization, and reconciliation action at the program level and/or institutional level; areas of action towards Indigenization, decolonization, and reconciliation; and systemic barriers and supports needed.

With respect to respondents’ characteristics, this study on the activities of dietetic education and training programs in Canada received a strong survey response rate of 69%. Moreover, characteristics of programs that responded to the survey reflected the landscape of dietetic education and training programs, with more programs located in Central Canada versus the West Coast and Prairie Provinces, or Atlantic Provinces and a higher percentage being undergraduate degrees versus discrete post-undergraduate degree practicums or combined masters practicum programs. Failure to translate the survey to French may have influenced the response rate in Francophone provinces. For example, in Quebec and New Brunswick, only 50% of the programs (2 of 4 and 1 of 2, respectively) completed the survey, versus 74% of programs in the rest of Canada.

Indigenization, decolonization, and reconciliation action at the program level and/or institutional level indicates that the healthcare-related TRCC Calls to Action have high relevance to dietetics. Specifically:

- Call #22 “*recognize the value of Aboriginal healing practices*” (which, in the case of dietetics, might encompass traditional foods).
- Call #23. i “*increase the number of Aboriginal professionals working in the health-care field.*”
- Call #23. iii “*provide cultural competency training for all health-care professionals.*”
- Call #24 “*require all students to take a course dealing with Aboriginal health issues, including the history and legacy of residential schools, the United Nations Declaration on the Rights of Indigenous Peoples, Treaties and Aboriginal rights, and Indigenous teachings and practices. This will require skills-based training in intercultural competency, conflict resolution, human rights, and anti-racism*” (TRCC, 2015).

At the time of the survey implementation (2022), most programs had implemented some changes to curricula and cultural immersion practices in response to these Calls to Action. Furthermore, most institutions provided some cultural safety and/or anti-racism training to staff and faculty and had implemented both Indigenous advisory councils/boards and Indigenization, decolonization, and reconciliation policies.

The activities of the institutions hosting dietetic education and training programs to integrate the Calls to Action identified through this survey seem comparable to what other academic institutions are doing across Canada. According to a Universities Canada 2022 survey, 96% of institutions reported offering training and resources for non-Indigenous students, faculty, staff, and leadership to support their responsibility to work towards reconciliation, while 72% reported having a partnership with a local Indigenous community (Universities Canada, 2023). The observed division of responsibilities between dietetic programs and their institutions towards addressing the Calls to Action may reflect the governance of dietetic programs in Canada; curricula and cultural immersion practices are predominantly tailored to each accredited program with institutional support.

Regarding areas of action towards Indigenization, decolonization, and reconciliation, more than 50% of respondents identified having strategies to attract and support Indigenous students or interns (Call #23). Targeted recruitment processes have also been increasingly adopted by other health professions across Canada. A strong example is the 2020 Joint Commitment to Action on Indigenous Health of Canadian medical schools to “*work towards admitting a school specific minimum number of First Nations, Métis and Inuit students each year by employing distinctions-based approaches and practicing holistic file reviews*” (Association of Faculties of Medicine of Canada, 2022; p. 6). By the time of their 2022 review, strategies within medicine included hiring an Indigenous Recruitment and Student Support Coordinator and offering a pre-admissions workshop for Indigenous applicants. Nevertheless, barriers such as financial constraints or the existence of unsupportive learning and training environments can affect Indigenous Peoples’ choice to apply to or complete a healthcare program (Burm et al., 2023; Gibson et al., 2022). The same factors likely affect the recruitment and retention of Indigenous dietetic students and trainees.

Reflecting on action towards addressing Call #24, 90% respondents reported having Indigenous-focused courses, incorporating some course content on Indigenous Peoples or offering lectures led by Indigenous Peoples. This is consistent with current activities in the education and training of other healthcare professionals, some of which go beyond Indigenous content, to processes of creating curricula (with an emphasis on co-creation with Indigenous Peoples) and its evaluation (Godwin et al., 2023; Melro et al., 2023; Sauvé et al., 2022). However, this survey did not assess the depth and breadth of curriculum coverage (i.e., number of Indigenous-focused courses or the “amount” of content within courses). Our findings that less than a third of respondents have mandated Indigenous-focused courses (or courses with content on Indigenous Peoples) is concerning. This is an example of how current curricula activities may not be effectively advancing processes of Indigenization, decolonization, and reconciliation.

Activities to enhance curricula were diverse in the current study, as has been observed elsewhere (Francis-Cracknell et al., 2019; Godwin et al., 2023; Melro et al., 2023; Sauvé et al., 2022). However, the current survey did not evaluate the impacts of curricula enhancement activities. Research suggests that activities may enhance cultural awareness more than Indigenous cultural safety and security (Francis-Cracknell et al., 2019). In one instance, an outcome of a 4- to 6-week instructional module about Indigenous Peoples and effects of colonization for first-year health professional students was unexpected; pre-post surveys over 3 years of administering the course found that one of the student cohorts (*n* = 154 of 355 respondents) increased scores for attitudes towards blaming Indigenous Peoples for their health inequities and decreased scores indicating agreement with government social action and policy to support Indigenous Peoples (Melro et al., 2023). These unexpected changes were associated with higher pre-course blaming attitudes and were more dominant among males and those from a program with low emphasis on social determinants of health (Melro et al., 2023). As Stein et al. point out, the lack of attention that dietetic programs currently give to social justice advocacy and the fact that they primarily operate within colonial institutions emphasizing Eurocentric views increase the risk of transforming activities towards curricula enhancement into mere items to “check off” a list that is not rooted in a systemic approach to Indigenization, decolonization, and reconciliation (Stein et al., 2023). Programs are encouraged to ensure that Indigenization activities are integrated in a program-wide, strategic approach to addressing the TRCC Calls to Action, including training on cultural safety for all students, staff, and faculty. Given that lack of resources was identified by respondents as the main barrier to advancing Indigenization, decolonization, and reconciliation, dietetic programs should also consider the potential for collaborative education approaches across Canada while leaving the opportunity for program-level adaptations that incorporate the uniqueness of local Indigenous communities.

About 80% of respondents indicated cultural immersion practices have been introduced, with the most common practice reported being placements within Indigenous settings. Offering such placements is common to other healthcare education and training programs (Francis-Cracknell et al., 2019; McDonald et al., 2018). A mixed methods study of dietetic graduates in Australia found that those who had completed an Indigenous placement reported significantly higher self-confidence when working with Indigenous Peoples (Svarc et al., 2018). A systematic review of Indigenous placements for medical, nursing, and allied health students, also in Australia, further added that this practice “*increased understanding and awareness of Aboriginal culture, promoted deeper understanding of Aboriginal health determinant complexity, increased awareness of everyday racism toward Aboriginal Australians, and enhanced desire to work in Aboriginal health*” (McDonald et al., 2018, p. 154). These outcomes of Indigenous placements were shown to have a lasting impact well into the early years of practice of a cohort of midwives in Australia (Thackrah et al., 2020). It is unknown whether placements translate into an increase in cultural safety in the healthcare provided to Indigenous Peoples across various settings (Hardy et al., 2023; MacLean et al., 2023). Therefore, dietetic programs should not only evaluate the impact of Indigenous placements on participating students, but also consider the effects on the Indigenous communities or Peoples served. Feedback from respondents and the healthcare education and training literature shows that it can be hard to access Indigenous placements for all students for a range of reasons (Ansell, 2022). Given the importance of these experiences, the University of British Columbia Dietetics program has implemented the *Dietetics Rural and Indigenous Communities Placement Fund*, a fundraising campaign to provide financial support for students completing placements in rural and Indigenous communities (University of British Columbia, 2023).

In line with the demands of Call to Action #23.iii, 85% of dietetic programs reported extending Indigenization, decolonization, and reconciliation activities to their staff and faculty by providing cultural safety training at the institutional level. A recent scoping review described the development, implementation, and evaluation of the different Indigenous-focused cultural training approaches (MacLean et al., 2023). Their effectiveness in increasing cultural safety for Indigenous Peoples is not confirmed (Hardy et al., 2023; MacLean et al., 2023). The approach to cultural safety training for programs that responded to the current survey was primarily non-mandatory webinars. A broader array of cultural safety training approaches, preferably in collaboration with Indigenous Peoples, is recommended (MacLean et al., 2023). In addition, dietetic programs should consider evaluating their cultural safety training approaches with a focus on patient-related satisfaction and outcomes (Hardy et al., 2023; MacLean et al., 2023).

Lack of resources (e.g. funding, staff, time), knowledge, and diversity (i.e., cultural and gender diversity within faculty) were main barriers to advancing Indigenization, decolonization, and reconciliation identified by respondents. Similar barriers were noted by Doria et al. (2021), who also indicated a limited and overburdened number of Indigenous faculty and competing priorities in the curriculum (Doria et al., 2021). As discussed above, one approach to mitigate key barriers may lie in dietetic programs’ collective capacity. Respondents felt that such “coming together” to advocate for academic resources and advance Indigenization, decolonization, and reconciliation could be facilitated by the broader Canadian dietetic leadership, such as DC and the Alliance of Canadian Dietetic Regulatory Bodies. An example of this dynamic is Dietitians Australia’s commitment to reconciliation with Aboriginal and Torres Strait Islander Peoples through the development and ongoing implementation of a Reconciliation Action Plan (RAP) (Dietitians Australia, 2022). The RAP not only promotes knowledge and respect for Aboriginal and Torres Strait Islander Peoples among Dietitians Australia staff and members, but it also provides a framework for its reconciliation activities (Dietitians Australia, 2022). The continuous actions of the RAP working group have led to important achievements since 2017, including the development of guidelines for a strengths-based approach to Aboriginal and/or Torres Strait Islander terminology; the assessment of Dietitians Australia cultural capability needs to inform future cultural safety training; engagement with other dietetic institutions to provide input into the National Competency Standards for dietitians and the dietetic education programs’ accreditation standards (Dietitians Australia, 2022); and a practical guide for dietetic educators (Murray et al., 2023). The guide includes five “signposts” to support dietetic programs in rooting their actions in the foundational elements of reconciliation and existing best practices, while also encouraging personal development and engagement through self-reflection and humility (Murray et al., 2023). Dietetic leadership in Canada can benefit from Dietitians Australia ongoing work and frameworks to help dietetic education and training programs advance Indigenization, decolonization, and reconciliation.

### Strengths and limitations

Key strengths of this survey are the high response rate (69%) and description of 2022 practice against a comprehensive list of Indigenization, decolonization, and reconciliation activities. This broad portrayal of activities taking place within Canadian dietetic education and training programs and institutions serves as a benchmark for future activities to advance the TRCC Calls to Action (TRCC, 2015). However, a few important limitations need to be considered. For instance, the survey was only provided in English, which may have influenced the non-participation of four dominantly Francophone dietetic education and training programs. Also, Indigenization, decolonization, and reconciliation in healthcare education is a rapidly evolving area and the results of the survey represent only a snapshot in time for 2022. Finally, survey results relied on respondents’ familiarity with the work of their program and/or institution and lacked details on the depth and breadth of involvement in Indigenization, decolonization, and reconciliation.

## Conclusion

This study is the first to describe Canadian dietetic education and training activities to address the TRCC Calls to Action. Our findings show that activities are shared between programs and host institutions, with programs steering the Indigenization of curricula and cultural immersion practices, two of the most frequently adopted activities. Attracting and supporting Indigenous students, interns, staff, and faculty, and providing cultural safety training to staff and faculty were also commonly adopted activities, including prioritizing Indigenous students within the admissions process. However, lack of resources (i.e., funding, staff, time) to support Indigenization, decolonization, and reconciliation was identified as a systemic barrier, along with a call for increased support from dietetic leadership. Evidence indicates that Canadian dietetic education and training programs activities are consistent with other healthcare professions. Yet, the lack of comprehensive mandatory curriculum for all dietetic students/interns and the lack of comprehensive, mandatory Indigenous cultural safety training for all students, staff, and faculty indicate that there is still work to do. Further action should focus on (1) evaluating current and future Indigenization, decolonization, and reconciliation activities; (2) reviewing current activities and ensuring they are part of a comprehensive approach to Indigenization, decolonization, and reconciliation, guided by Indigenous Peoples and rooted in Indigenous social justice and health equity principles; and (3) exploring the adoption of collaborative action and advocacy as tools to overcome the systemic barriers to advance Indigenization, decolonization, and reconciliation, with dietetic leadership’s support. Canadian dietetic leadership has, in Dietitians Australia’s Reconciliation Action Plan, a comprehensive set of tools and frameworks to inform the profession’s approach to Indigenization, decolonization, and reconciliation.

## Contributions to knowledge

What does this study add to existing knowledge?

- The TRCC (2015) advocates for action within Canadian healthcare education. There has been no previous comprehensive inventory of activities within dietetics.
- This study provides:
  - an inventory of Canadian dietetic education and training programs’ activities towards Indigenization, decolonization, and reconciliation in 2022;
  - perspective into the systemic barriers and needed support for programs to advance Indigenization, decolonization, and reconciliation; and
  - awareness of challenges within Indigenization, decolonization, and reconciliation action, and ways to address such challenges, ideas, and resources that can support programs (dietetic or other healthcare professions) in their work.

What are the key implications for public health interventions, practice, or policy?

- More action to address the TRCC Calls to Action is needed.
- Programs are encouraged to evaluate their current and future activities.
- Indigenization, decolonization, and reconciliation action has been criticized for its checklist approach. Programs are encouraged to integrate activities within a comprehensive approach rooted in Indigenous social justice and health equity principles.
- To overcome barriers such as lack of resources, programs are encouraged to come together in work and advocacy, supported by dietetic leadership (i.e., professional associations).
- Canadian dietetic leadership have in Dietitians Australia’s work a great framework to inform its own practice and policies.

## Supplementary Information

Below is the link to the electronic supplementary material.

Supplementary file1 (DOCX 45 KB)

Supplementary file2 (DOCX 31 KB)

## Data Availability

The data that support the findings of this study are available, upon reasonable request, from the corresponding author. The data are not publicly available due to containing information that could compromise the privacy of the study’s participants.
