# Supplementary material for: Canadian dietetic education and training actions to support Indigenization, decolonization, and reconciliation
Source: Can J Public Health. 2025 Jun 12;117(1):14–30. doi: 10.17269/s41997-025-01055-z (PMC12992849; doi:10.17269/s41997-025-01055-z)
Supplement: Supplementary file 1 — Supplementary file1 (DOCX 45 KB) [file 41997_2025_1055_MOESM1_ESM.docx]

**Supplementary file 1 – Survey**

**Caption:** a cross-sectional, online, self-administered survey distributed to dietetic education and training programs in Canada, to assess their efforts toward Indigenization, decolonization, and reconciliation.

**Article title:** Canadian dietetic education and training actions to support Indigenization, decolonization, and reconciliation

**Journal name:** Canadian Journal of Public Health

**Author names:** Laura Correia Dias*, Chelsea Leslie, Victoria Emmell, Rhona M. Hanning, Lee Rysdale, Sandra Juutilainen, Shannan Grant, Kelly Gordon, Hannah Neufeld

**Affiliation and e-mail address of the corresponding author: ***School of Public Health Sciences, Faculty of Health, University of Waterloo. Email: [l3dias@uwaterloo.ca](mailto:l3dias@uwaterloo.ca).

**Welcome to the *Current efforts to support Indigenization, decolonization, and reconciliation in Canadian dietetic education & training* survey.**

**Thank you in advance for your participation.**

A few notes and reminders before you start:

- The survey will take about 20 to 30 minutes to complete, depending on the number of actions that your program and/or institution has taken towards Indigenization, decolonization, and reconciliation. The questions are divided into three potential areas of action: education & training, professional practice, and strategic planning, policies & quality improvement. **Please note that your answers are being automatically saved as you progress in the survey. Upon completion of the survey, you will be given the opportunity to review your responses and download them in PDF format.**
- The survey will be open until **April 30, 2022**. During this time, you are able to complete the survey at your own pace. **You can save your progress and come back at any point.**
- Respondents must hold a position within a dietetic education and/or training program in Canada (as accredited by the Partnership for Dietetic Education and Practice). Ideally, they will also have knowledge of current initiatives and strategies taking place in said program and/or institution, with the goal of advancing Indigenization, decolonization, and reconciliation efforts.
- **We ask to have one survey completed per each program**. Therefore, if your role encompasses more than one program, please complete a separate survey for each one of the programs. If you are unable to respond to more than one survey OR are not the best person to complete this survey, please share with another knowledgeable staff member who can respond on behalf of your program.
- Consent will be requested in the next section. You may withdraw it at any time during the project, without any negative consequences, by emailing Laura Dias at [l3dias@uwaterloo.ca](mailto:l3dias@uwaterloo.ca). If, at the moment of consent withdrawal, you have completed the survey but no longer want the data to be included in the project, please indicate so in your email and include the following information: the dietetic program you oversee and your title within the program (as responded in the survey). This will allow the researchers to retrieve and delete all the answers you provided in the survey.
- **You may decline to answer any of the survey questions if you so wish, without any negative consequences**. You may also decide to end your participation at any time by simply closing your browser.
- While the survey will ask respondents to identify their title and their dietetic program, this information will be kept confidential in the published results. However, please keep in mind the data will be presented in aggregate (i.e., by province and/or by program type) which, given the limited number of programs in Canada, particularly in smaller provinces, means that there is a risk of certain programs being identified in the published results.
- This study has been reviewed and received ethics clearance through a University of Waterloo Research Ethics Board (REB #42577). If you have questions for the Board, contact the Office of Research Ethics at 1-519-888-4567 ext. 36005 or [reb@uwaterloo.ca](mailto:reb@uwaterloo.ca).

If you need more information or have any concerns about the survey, please contact Dr. Rhona Hanning at [rhanning@uwaterloo.ca](mailto:rhanning@uwaterloo.ca) (principal investigator) or Laura Dias at [l3dias@uwaterloo.ca](mailto:l3dias@uwaterloo.ca).

**Consent**

**Note**: By participating in this survey, you consent to provide data for use in the University of Waterloo-led research project *Towards Decolonizing Dietetic Practice in Canada*. A part of the larger project is this specific research survey conducted with dietetic interns from the Northern Ontario School of Medicine (NOSM) University Dietetic Practicum Program (DPP). By providing your consent, you are not waiving your legal rights or releasing the investigator(s) or involved institution(s) from their legal and professional responsibilities.

This study has been reviewed and received ethics clearance through a University of Waterloo Research Ethics Board (REB #42577). If you have questions for the Board, contact the Office of Research Ethics at 1-519-888-4567 ext. 36005 or [reb@uwaterloo.ca](mailto:reb@uwaterloo.ca).

For all other questions please contact Dr. Rhona Hanning at [rhanning@uwaterloo.ca](mailto:rhanning@uwaterloo.ca) (principal investigator) or Laura Dias (graduate student) at [l3dias@uwaterloo.ca](mailto:l3dias@uwaterloo.ca).

**(Required response to all questions)**

1. I have read the information presented in the information letter about the project being conducted by NOSM University Dietetic Practicum Program (DPP) dietetic interns Chelsea Leslie, and Victoria Emmell, under supervision of Lee Rysdale (DPP), Rhona Hanning (UW) and Laura Dias (UW).

- Yes
- No

2. I have been given the opportunity to ask any questions related to this research project, and, if so, to receive satisfactory answers to my questions, and any additional details I wanted.

- Yes
- No

3. I agree, of my own free will, to participate in this survey, knowing that I can withdraw at any point with no consequences to me.

- Yes
- No

4. I agree with the use of anonymous data, aggregated by province and/ or program type, in any reports, presentations or publications resulting from the DPP project and the larger *Towards Decolonizing Dietetic Practice in Canada* project.

- Yes
- No

5. I agree to the use of anonymous quotations from my responses on any publications resulting from this project.

- Yes
- No

**Program information**

**Note**: The questions in this section focus on the dietetic program you oversee/work for. **We ask that you select only one program and respond to the survey based on that program**.

If you oversee/work for more than one dietetic program (i.e., undergraduate program and post degree practicums), we ask that you complete a separate survey for each program (or have another staff completing the survey for the other program).

1. Please select the dietetic program you oversee. **(Required response)**

**Academic Education and Practicum Training**

- University of British Columbia, Food, Nutrition and Health Program (BSc), Dietetics Major
- University of Alberta, Bachelor of Science in Nutrition & Food Science, Dietetics Specialization
- University of Saskatchewan, Bachelor of Science in Nutrition
- Université d’Ottawa, Baccalauréat spécialisé en sciences des aliments et de la nutrition
- McGill University, Bachelor of Science in Nutrition, Dietetics Major
- Université de Montréal, Baccalauréat en nutrition
- Université Laval, Baccalauréat en nutrition (B.Sc.)
- Université de Moncton, Baccalauréat en nutrition
- Prefer not to disclose specific program, but it is an undergrad degree with a fully integrated practicum

**Undergraduate Degrees: Partially Integrated**

- Acadia University, Bachelor of Science in Nutrition (Dietetics option)
- Acadia University, Dietetic Practicum Program
- Mount Saint Vincent University, Bachelor of Science in Applied Human Nutrition (Dietetics)
- Mount Saint Vincent University, BScAHN (Dietetics) Internship Education Program
- St. Francis Xavier University, Human Nutrition Program
- St. Francis Xavier University, Integrated Dietetic Internship
- University of Prince Edward Island, Bachelor of Sciences (Foods and Nutrition)
- University of Prince Edward Island, Integrated Dietetic Internship
- Prefer not to disclose specific program, but it is an undergrad degree with a partially integrated practicum

**Undergraduate Degrees: Not Integrated**

- University of Manitoba, Bachelor of Science in Human Nutritional Sciences
- Brescia University College, Bachelor of Science, Honors Specialization in Nutrition and Dietetics
- Ryerson University, Nutrition and Food (BASc) Program
- University of Guelph, Bachelor in Applied Human Nutrition
- Prefer not to disclose specific program, but it is a non-integrated undergrad degree

**Post Degree Practicums**

- Winnipeg Regional Health Authority, Manitoba Partnership Dietetic Education Program
- Brescia University College, Diploma in Dietetic Education and Practical Training
- Northern Ontario School of Medicine, Northern Ontario Dietetic Internship Program (NODIP)
- Ryerson University, Professional Masters Diploma in Dietetics Program (PMDip)
- Nova Scotia Health Authority, Central Zone Dietetic Internship Program
- The Moncton Hospital, Graduate Internship Dietetic Program
- Eastern Health, Dietetic Internship Program (also provides internship for Memorial University Master of Public Health – Nutrition/Dietetics Stream)
- Prefer not to disclose specific program, but it is a post-degree practicum

**Masters Education with Practicum Training**

- Brescia University College, Master of Science in Food and Nutrition (Internship Stream)
- Ryerson University, Master of Health Science in Nutrition Communication
- University of Guelph, Master of Applied Nutrition
- University of Toronto, Master of Public Health, Nutrition and Dietetics
- McGill University, Master of Science Applied Program in Human Nutrition (Dietetics Credentialing)
- Mount Saint Vincent University, Master of Science Applied Human Nutrition with Internship Education Program
- Prefer not to disclose specific program, but it is a graduate program with fully integrated practicum

2. What is your role within this particular program? Select all that apply.

- Program director/manager
- Internship coordinator
- Regional coordinator
- Professor/instructor
- Other. **Please elaborate**:

____________________________________________________________________________________

3. What is the size of your program? (Average number of new students admitted each year)

____________________________________________________________________________________

4. Are you a Registered Dietitian?

- Yes
- No
- Other. Please elaborate: _____________________________________________________________

**MAIN MATRIX QUESTION**

**Definitions**

**Indigenization**: “Efforts to transform spaces, processes and institutions founded in non-Indigenous cultures to include Indigenous ways of knowing, being, and acting and the persons who practice them.” (p.149)

**Decolonization**: Involves undoing colonial influences. For settlers, this means acknowledging and reversing internalized racist or discriminatory viewpoints; learning and drawing from Indigenous culture and knowledges; respecting and enabling the self-determination of Indigenous peoples. (p.142)

**Reconciliation**: “Efforts to create mutually respectful relationships between Indigenous and non-Indigenous Peoples, or to benefit in terms of power and status from the appearance of such efforts.” (p.150)

Reference: Grafton, E., & Melançon, J. (2020). The dynamics of decolonization and Indigenization in an era of academic “reconciliation”. In Cote-Meek, S., & Moeke-Pickering, T. (Eds.), Decolonizing and Indigenizing education in Canada. (pp. 135 - 153). Canadian Scholars.

1. Within your program, what areas of action are you working on towards Indigenization, decolonization, and/or reconciliation? (Select all that apply)

|  | Yes, at the  program level* | Yes, at the  institutional level | No |
| --- | --- | --- | --- |
| **Education and Training** |  |  |  |
| A. Attracting and supporting Indigenous Peoples (students/interns, staff & faculty, preceptors) |  |  |  |
| B. Curriculum content on Indigenous Peoples** |  |  |  |
| C. Cultural Immersion practices (i.e., practicum placements in Indigenous communities) |  |  |  |
| **Professional Practice** |  |  |  |
| D. Cultural safety and or anti-racism training for staff & faculty |  |  |  |
| E. Partnership with Indigenous Peoples (i.e., access to Elders/ Knowledge Keepers) |  |  |  |
| **Strategic Planning, Policies, Quality Improvement** |  |  |  |
| F. Strategic planning |  |  |  |
| G. Designated advisory board/council |  |  |  |
| H. Policies regarding Indigenization, decolonization, and reconciliation |  |  |  |
| I. Quality improvement (i.e., evaluation of current practices) |  |  |  |

**(For sections A to I, only reply to the ones to which you answered “Yes” in this matrix question)**

* Includes actions in planning stages.
** As described in the 2020 Integrated Competencies for Dietetic Education and Practice (ICDEP). Available at <https://www.pdep.ca/library/PDEP-Policies/Integrated-Competencies-For-Dietetic-Education-And.aspx>

**A. Attracting and supporting Indigenous Peoples (students/interns, staff & faculty, preceptors)**

**STUDENTS & INTERNS**

1. Are there approaches in place to attract and support Indigenous students/ interns to your program/ institution?

- Yes **(skip to question 2)**
- No **(skip to question 3)**
- Not sure. **Please elaborate**: **(skip to question 2)**

_____________________________________________________________________________________

2. Please select all approaches currently in place.

If possible, **please briefly elaborate** on the approaches selected (i.e., number of spots, type of academic support).

- Prioritizing spots for Indigenous students/interns _____________________________________________________________________________________
- Financial support (i.e., grants, waived application fees) _____________________________________________________________________________________
- Improving access to Indigenous mentors (i.e., Indigenous dietitians, Indigenous preceptors, Indigenous faculty, Indigenous staff)

_____________________________________________________________________________________

- Offering living/meetings spaces & events for Indigenous students/interns (i.e., Indigenous Student Centre)

_____________________________________________________________________________________

- Dedicated academic support (i.e., ensuring academic advisors have cultural safety training) _____________________________________________________________________________________
- Supporting access to personal or cultural supports (i.e., access to Elders/ Knowledge Keepers, culturally sensitive counseling support)

_____________________________________________________________________________________

- Increased accessibility (i.e., online learning for students from remote areas) _____________________________________________________________________________________
- Community outreach to Indigenous high school students _____________________________________________________________________________________
- Other. **Please elaborate**:

_____________________________________________________________________________________

**STAFF & FACULTY**

**Note**: the definition of staff used for this section includes administration staff, program managers/directors, instructors, and professors. Preceptors are not included.

3. Are there approaches in place to attract and support Indigenous staff & faculty in your program/ institution?

- Yes **(skip to question 4)**
- No **(skip to question 5)**
- Not sure. **Please elaborate**: **(skip to question 4)**

_____________________________________________________________________________________

4. Please select all approaches currently in place.

If possible, **please briefly elaborate** on the approaches selected (i.e., types of professional development supported).

- Prioritizing spots for Indigenous staff & faculty _____________________________________________________________________________________
- Financial incentives (i.e., additional health benefits) _____________________________________________________________________________________
- Dedicated spaces & events for Indigenous staff _____________________________________________________________________________________
- Professional development support (i.e., supporting costs of conference attendance) _____________________________________________________________________________________
- Supporting access to personal or cultural supports (i.e., access to Elder/ Knowledge Keeper, counseling services with cultural safety training, peer supports) _____________________________________________________________________________________
- Other. **Please elaborate**:

_____________________________________________________________________________________

**PRECEPTORS**

5. Are there approaches to support student access to Indigenous preceptors in your program/institution? (i.e., established relationships with the program, supporting placements in student's own community settings)

- Yes. **Please briefly elaborate** on the types of approaches: _____________________________________________________________________________________
- No
- Not applicable
- Not sure. **Please elaborate**:

_____________________________________________________________________________________

6. Aside from what has been mentioned, are there other ways in which your program/institution is working towards attracting and supporting Indigenous students/interns, staff & faculty, and preceptors?

- Yes. **Please elaborate**:

_____________________________________________________________________________________

- No

**B. Curriculum content on Indigenous Peoples**

1. How is content on Indigenous Peoples in Canada incorporated in the program curriculum? Select all that apply.

If possible, **please briefly elaborate** on the approaches selected (i.e., number of courses, frequency of guest lectures)

- Undergraduate, graduate or internship course(s) or course content on Indigenous Peoples **(skip to question 2)**

_____________________________________________________________________________________

- Guest lectures by Indigenous Peoples (i.e., Elders, sharing circles) **(skip to next section where YES was selected)**

_____________________________________________________________________________________

- Field trips (i.e., to an Indigenous cultural centre) **(skip to next section where YES was selected)**

_____________________________________________________________________________________

- One-time events (i.e., conferences, webinars) **(skip to next section where YES was selected)**

_____________________________________________________________________________________

- Course work (i.e., assigned readings, assignments) **(skip to next section where YES was selected)** _____________________________________________________________________________________
- Practicum placements **(skip to next section where YES was selected)**

_____________________________________________________________________________________

- Land-based learning **(skip to next section where YES was selected)**

_____________________________________________________________________________________

- Other. **Please elaborate**: **(skip to next section where YES was selected)**

_____________________________________________________________________________________

2. For the courses on Indigenous Peoples in Canada (or with content on Indigenous Peoples in Canada), please describe their designation.

- Mandatory
- Elective
- Other. **Please elaborate**:

_____________________________________________________________________________________

3. What topics are usually covered in the courses/course content on Indigenous Peoples in Canada? Select all that apply.

- Indigenous Values and ways of knowing related to health and wellness (i.e., Traditional foods & Indigenous foodways)
- Historic and ongoing impacts of colonization, Indian residential schools, intergenerational trauma, systemic racism (i.e., nutrition experiments associated with Indian Residential Schools; systemic racism in health care settings)
- Past & ongoing nutrition inequities
- Social determinants of health
- Others. **Please elaborate**:

_____________________________________________________________________________________

**C. Cultural immersion practices**

**Note**: cultural immersion practices, also known as intentional placements, are activities where students/interns can immerse in the culture of Indigenous Peoples in Canada. They include practicum placements or week-long camps. They fit under the concept of wise practices, defined as “locally-appropriate actions, tools, principles or decisions that contribute significantly to the development of sustainable and equitable conditions” (Calliou and Wesley-Esquimaux, 2010, p.19).
 
Reference: Wesley-Esquimaux, C., & Calliou, B. (2010). Best practices in Aboriginal community development: A literature review and wise practices approach. The Banff Centre, 1-38. Retrieved from <http://communities4families.ca/wp-content/uploads/2014/08/Aboriginal-Community-Development.pdf>

1. Are these cultural immersion practices available for all students/interns?

- Yes, and they are required for all students/interns
- Yes, but they are not required for all students/interns
- No, there is no capacity to provide these practices for all students
- Other. **Please elaborate**:

_____________________________________________________________________________________

2. What type of cultural immersion practices are offered to students/interns through your program/institution?

If possible, **please briefly elaborate** on the practices selected (i.e., length of placement)

- Practicum placements/rotations in Indigenous communities/settings serving Indigenous Peoples (i.e., Friendship Centres)

_____________________________________________________________________________________

- One-time immersion opportunities (i.e., one week camp, weekend in an Indigenous community setting)

_____________________________________________________________________________________

- Other. **Please elaborate**:

_____________________________________________________________________________________

3. Does your program/institution offer preparation to students/interns, in advance of their cultural immersion opportunities? (i.e., cultural safety training, lecture, workshop)

- Yes. **Please describe**:

_____________________________________________________________________________________

- No
- Other. **Please elaborate**:

_____________________________________________________________________________________

4. Does your program/institution offer the opportunity for self-reflection/debriefing to students/interns who completed a cultural immersion opportunity?

- Yes. **Please describe**:

_____________________________________________________________________________________

- No
- Other. **Please elaborate**:

_____________________________________________________________________________________

5. Aside from what has been shared above, is there anything you would like to add about cultural immersion practices in your program/institution?

- Yes. **Please elaborate**:

_____________________________________________________________________________________

- No

**D. Cultural safety and/or anti-racism training for staff & faculty**

**Note**: cultural safety and/or anti-racism training can be delivered in-person or online, and include university-wide professional development courses, webinars (internal/external), and courses/workshops administered by external organizations. Please note that, for the purpose of this survey, they must, at least, include a focus on Indigenous Peoples in Canada.

1. What type of cultural safety and/or anti-racism training does your program/institution provide to staff & faculty? Select all that apply.

If possible, **please briefly elaborate** on the type of training provided.

- Webinars (i.e., webinars for the National Day for Truth and Reconciliation) _____________________________________________________________________________________
- Courses (i.e., Indigenous Canada course from the University of Alberta [1]) _____________________________________________________________________________________
- Workshops (i.e., Blanket Exercise [2])

_____________________________________________________________________________________

- Other. **Please elaborate**:

_____________________________________________________________________________________

2. Is cultural safety and/or anti-racism training mandatory for staff & faculty in the program/institution? Select all that apply.

- Yes, as part of the orientation process
- Yes, as part of ongoing human resources requirements
- No
- Other. **Please elaborate**:

_____________________________________________________________________________________

[1] University of Alberta Faculty of Native Studies. (2021). Indigenous Canada. Retrieved from <https://www.ualberta.ca/admissions-programs/online-courses/indigenous-canada/index.html>  
[2] KAIROS Canada. (2020). KAIROS Blanket Exercise. Retrieved from <https://www.kairosblanketexercise.org/>

**E. Partnership with Indigenous Peoples**

1. Please select all types of existing external* partnerships with Indigenous Peoples communities and/or organizations in place in your program/institution, to support efforts towards Indigenization, decolonization, and reconciliation:

- For cultural immersion practices
- For curriculum enhancement
- For strategic planning
- For supportive environments for Indigenous students/interns, staff & faculty, and preceptors
- For ongoing support with program (i.e., Elder/ Knowledge Keeper on staff)
- Other. **Please elaborate**:

_____________________________________________________________________________________

* Separate from any work done with internal staff and faculty who identify as Indigenous.

**F. Strategic planning**

1. Does your program/institution have a strategic plan in place that includes a focus on Indigenization, decolonization and/or reconciliation?

- Yes, there is a strategic plan for the program only
- Yes, there is a strategic plan for the institution only
- Yes, there is a strategic plan for the program and the institution
- No, there is no strategic plan for the program nor the institution with this focus
- No, but it is being developed for our program only
- No, but it is being developed for our institution only
- No, but it is being developed for our program and institution
- Other. **Please elaborate**:

_____________________________________________________________________________________

**G. Designated advisory board/council**

1. Does your program/institution have an Indigenous Advisory council, board, or group?

- Yes, only for our program
- Yes, only for the institution
- Yes, and it is shared between the program and the institution
- Yes, one for the program and a different one for the institution
- No, and no plans to have one in the future.
- Not, but planning is underway to have one in the future.
- Other. **Please elaborate**:

_____________________________________________________________________________________

**H. Policies regarding Indigenization, decolonization, and reconciliation**

1. What is the focus of the policies regarding Indigenization, decolonization, and reconciliation currently in place in your program/institution? Select all that apply.

- Attracting and supporting Indigenous Peoples
- Identifying & eradicating racism against Indigenous students and staff
- Establishing and maintaining appropriate relationships with Indigenous partners
- Conducting research with Indigenous Peoples
- Providing of tobacco, gifts, or honorariums to Indigenous Peoples
- Other. **Please elaborate**:

_____________________________________________________________________________________

**I. Quality improvement**

1. Does your program/institution evaluate, on a regular basis, action taken towards Indigenization, decolonization, and reconciliation?

- Yes **(skip to question 2)**
- No **(skip to Assessment of candidates’ prior learning section)**
- Not sure. **Please elaborate** **(skip to Assessment of candidates’ prior learning section)**

_____________________________________________________________________________________

- Other. **Please elaborate**: **(skip to Assessment of candidates’ prior learning section)**

_____________________________________________________________________________________

2. Please select below all actions that are subject to evaluation/monitoring in your program:

- Attract and support Indigenous students/interns, staff & faculty, and preceptors
- Curriculum content on Indigenous Peoples in Canada
- Cultural Immersion practices
- Cultural safety and/or anti-racism training for staff & faculty
- Partnerships with Indigenous Peoples
- Strategic planning
- Indigenous Advisory groups
- Policies regarding Indigenization, decolonization, and reconciliation
- Incident reporting (i.e., racism towards Indigenous Peoples)
- Other. **Please elaborate**:

_____________________________________________________________________________________

**Assessment of candidates' prior learning**

1. Does your program have a mechanism to assess students/interns’ prior learning on Indigenization, decolonization, and reconciliation?

- Yes **(skip to question 2)**
- No **(skip to next section)**
- Other. **Please elaborate**: **(skip to question 2)**

_____________________________________________________________________________________

2. Based on your estimation, what proportion of the students admitted every year are already entering the program with previous knowledge and/or experience of Indigenization, decolonization, and reconciliation?

- 0-20%
- 21-40%
- 41-60%
- > 61%
- Unsure/Don't know

***Thank you for your patience. You are close to completing the survey.***

1. Is there anything that your program/institution is doing on this topic that we didn't ask about and you would like to add?

- Yes. **Please elaborate**:

_____________________________________________________________________________________

- No

2. Aside from your current progress towards Indigenization, decolonization, and reconciliation, what might you like to see your program/institution doing in the near future, on this topic?

____________________________________________________________________________________

____________________________________________________________________________________

____________________________________________________________________________________

3. What are the three main systemic barriers you see for your program/institution to progress when it comes to Indigenization, decolonization, and reconciliation?

____________________________________________________________________________________

____________________________________________________________________________________

____________________________________________________________________________________

4. How might dietetics leadership in Canada (Dietitians of Canada, regulatory bodies, Partnership for Dietetic Education and Practice) help you advance Indigenization, decolonization, and reconciliation in your program/institution?

**Please provide one or two suggestions**.

____________________________________________________________________________________

____________________________________________________________________________________

____________________________________________________________________________________

5. Do you have any final thoughts to share? If yes, please add them below:

____________________________________________________________________________________

____________________________________________________________________________________

____________________________________________________________________________________

***Thank you • Mîkwêc • Naqumik • Nakumik • Quianamik • Mutna* •**

***Anushik • Marsi • Niá:wen • Miigwech****

The survey is now completed. We are very appreciative of the time you have taken to support our project and for the valuable information you have provided. We believe that the results of this survey will play an important role in advancing our collective efforts towards Indigenization, decolonization, and reconciliation in Canadian dietetic education & training.

**Next steps**

The data collected in this survey will be analyzed by the end of March 2022. The final results will be released throughout Spring 2022. This includes a presentation at the Dietitians of Canada 2022 Conference and to the Dietetic Educators Network. 
 
*Would you like to be contacted by the research team after the survey to receive study results, updates about the project, and/or to participate in additional components related to this study or new studies?*
  
*If yes, click on the link below (please note that you will not lose the answers you already completed). It will open a new, separate window where you can select for which purpose you want us to contact you about and where you can provide your email. This information will be stored separately from the responses you provided in the survey.*
  
*Once you complete this form, don't forget to return to the survey page to complete your submission.*

<https://uwaterloo.ca1.qualtrics.com/jfe/form/SV_08Q50moNjmLLjo2>

**Contact information**

If you have any questions about this survey or the overall DPP research project, please contact Laura Dias at [l3dias@uwaterloo.ca](mailto:l3dias@uwaterloo.ca).

*Retrieved from <http://www.copahabitat.ca/sites/default/files/languagetool.pdf>
