# Supplementary material for: Canadian dietetic education and training actions to support Indigenization, decolonization, and reconciliation
Source: Can J Public Health. 2025 Jun 12;117(1):14–30. doi: 10.17269/s41997-025-01055-z (PMC12992849; doi:10.17269/s41997-025-01055-z)
Supplement: Supplementary file 2 — Supplementary file2 (DOCX 31 KB) [file 41997_2025_1055_MOESM2_ESM.docx]

**Supplementary file 2 – Open-ended feedback**

**Caption:** Summary of the open-ended feedback (paraphrased) qualitative data on work being completed by dietetic education and training programs in Canada, towards Indigenization, decolonization, and reconciliation.

**Article title:** Canadian dietetic education and training actions to support Indigenization, decolonization, and reconciliation

**Journal name:** Canadian Journal of Public Health

**Author names:** Laura Correia Dias*, Chelsea Leslie, Victoria Emmell, Rhona M. Hanning, Lee Rysdale, Sandra Juutilainen, Shannan Grant, Kelly Gordon, Hannah Neufeld

**Affiliation and e-mail address of the corresponding author: ***School of Public Health Sciences, Faculty of Health, University of Waterloo. Email: [l3dias@uwaterloo.ca](mailto:l3dias@uwaterloo.ca).

| **EDUCATION AND TRAINING** |
| --- |
| **Section A – Attracting and supporting Indigenous Peoples (students/interns, staff & faculty, preceptors)** |
| 1. **Approaches to attract and support Indigenous students/ interns** |
| Prioritizing spots for Indigenous students/interns   - Indigenous applicants are automatically qualified for an interview. - Two spots are guaranteed for Indigenous students whose application meets the program admissions requirements. - A percentage of spots for research-focused programs are reserved for Indigenous students. - Other strategies within the admissions process:   - The scoring system favours applicants who self-identify as Indigenous.   - The admission process is blinded, to improve equity, diversity and inclusion.   - Indigenous students are asked to self-identify so the program can reach out to discuss opportunities (i.e., placements). |
| Financial support (i.e., grants, waived application fees)   - At the program and/or institution level (through their Indigenous departments) - Grants from Indigenous organizations (i.e., [INDSPIRE Scholarship](https://indspire.ca/programs/students/bursaries-scholarships/)) - Provincial government grants for Indigenous applicants |
| Improving access to Indigenous mentors (i.e., Indigenous dietitians, Indigenous preceptors, Indigenous faculty, Indigenous staff)   - Indigenous faculty within the program (often focusing on Indigenous health and nutrition) - Indigenous faculty at the institutional level - Partnerships with local Indigenous organizations are important to facilitate access to Indigenous mentors. - Access is provided if requested by a student/intern - It can be challenging to provide access given the small number of Indigenous preceptors. |
| Dedicated academic support (i.e., ensuring academic advisors have cultural safety training)   - Academic advisors and/or counselours who self-identify as Indigenous |
| Supporting access to personal or cultural supports (i.e., access to Elders/ Knowledge Keepers, culturally sensitive counseling support)   - At the institutional level (through their Indigenous spaces/departments) - Partnerships with local Indigenous communities to provide culturally safe spiritual care - Transition programs are available for first year Indigenous students. |
| Increased accessibility (i.e., online learning for students from remote areas)   - Driven by COVID (i.e., complete a portion of a placement online) - Through the institutional accessibility services - Can be impacted by institutional resistance (i.e., preference for in-person teaching) |
| Community outreach to Indigenous high school students   - Program director-led recruitment in schools within racialized and Indigenous communities - At the institutional level, often through events targeting Indigenous youth, led by local Indigenous organizations and/or the institution (i.e., summer camps) |
| 1. **Approaches to attract and support Indigenous staff and/or faculty** |
| Prioritizing spots for Indigenous staff & faculty   - Hiring period/focus/spots for Indigenous faculty, at the university level |
| Professional development support (i.e., supporting costs of conference attendance)   - Support to attend professional development events (i.e., conferences) - Available within the institutional (i.e., Indigenous Speaker series, Indigenous advisor) - Available for all staff, subject to application and approval (not exclusive for Indigenous staff) |
| Supporting access to personal or cultural supports (i.e., access to Elder/ Knowledge Keeper, counseling services with cultural safety training, peer supports)   - Through Indigenous spaces/departments within the institution - At the program level, can rely on faculty and/or students linked to local Indigenous communities |
| 1. **Approaches to support student access to Indigenous preceptors** |
| - Collaboration with Indigenous dietitians working within Indigenous communities - Securing placements with Indigenous dietitians - Collaboration with Indigenous organizations/communities (often local) - Working with Indigenous students to identify culturally safe placements within Indigenous communities - Connecting students with other relevant services at the institutional level (i.e., Indigenous Health department) - Procuring placements within Indigenous settings if requested by a student - The number of Indigenous dietitians is limited, which impacts the ability of securing Indigenous dietitians as preceptors. |
| 1. **Other ways to attract and support Indigenous students/interns, staff & faculty, and preceptors (institutional level)** |
| - Establishing Indigenous student cohorts - Hiring an Indigenous curriculum consultant - Hiring a regional Indigenous relations coordinator |
| **Section B – Curriculum content on Indigenous Peoples** |
| 1. **Approaches to incorporate Indigenous Peoples in Canada content in the program curriculum** |
| Undergraduate, graduate or internship course(s) or course content on Indigenous Peoples   - Content is scaffolded throughout the program curriculum. - Having Indigenous-focused courses (i.e., Indigenous Food Systems) - In research-based courses, students can select Indigenous-focused projects. - Requiring students/interns required to complete external Indigenous-focused programs/courses/workshops (i.e., [*Food is Our Medicine*](https://www.nourishleadership.ca/fiom-overview) course from [Nourish Leadership](https://www.nourishleadership.ca/about-1)) |
| Guest lectures by Indigenous Peoples (i.e., Elders, sharing circles)   - Facilitated by the programs’ Indigenous staff/faculty - Facilitated by the institutions’ Indigenous staff/faculty - Facilitated by Indigenous Peoples external to the program/institution - Can take the shape of pre-recorded lectures |
| One-time events (i.e., conferences, webinars)   - One-time events (often offered by the institution) are promoted to the students. - Events can be associated to Indigenous programs related to the institution or to local Indigenous communities. - Interns are required to attend cultural safety and diversity-related webinars. - Can also take the shape of a workshop. |
| Course work (i.e., assigned readings, assignments)   - Case studies; reflections; Indigenous content prepared outside the program/institution; readings |
| Land-based learning   - Partnership with local Indigenous organizations/communities to visit their programs (i.e., community gardens) |
| Other. Please elaborate:   - Content on Indigenous Peoples in Canada can also be incorporated in the curriculum by adding it to the orientation process. |
| 1. **Nature of Indigenous Peoples in Canada courses/with content about Indigenous Peoples in Canada (mandatory/not mandatory)** |
| - There is a mix of mandatory and elective courses focusing on Indigenous Peoples OR with Indigenous Peoples content. - Can be mandatory for students completing a dietetic stream, within the program. |
| 1. **Topics usually covered in the Indigenous Peoples in Canada courses/with content about Indigenous Peoples in Canada** |
| - Indigenous Peoples, cultural safety and the role of dietitians working with Indigenous Peoples across Canada. - Indigenous food sovereignty - Indigenous agriculture |
| **Section C - Cultural Immersion practices (i.e., practicum placements in Indigenous communities)** |
| 1. **Cultural immersion practices availability for students/interns** |
| - Interns can choose between an Indigenous placement or a Francophone placement. |
| 1. **Type of cultural immersion practices offered to students/interns** |
| Placements in Indigenous communities or settings serving Indigenous Peoples (i.e., Friendship Centres)   - Some placements are longer (4-8 weeks) while others are shorter (2-3 weeks). - Examples of placement settings: [Aboriginal Health Access Centres](https://www.ontario.ca/page/aboriginal-health-access-centres), Indigenous Health Authorities |
| 1. **Availability of preparation to students/interns in advance of their cultural immersion opportunities** |
| - Preparation is offered at the beginning of or throughout the program (not exclusive for an Indigenous community/setting placement). - One program has a mandatory Indigenous cultural safety training before Indigenous community/settting placement. - Another program has a policy/guideline in place to support interns in their placements within Indigenous communities/settings. - Preparation is often supported by the program directors, in collaboration with Indigenous community members (i.e., Elders). |
| 1. **Availability of opportunities for self-reflection/debriefing to students/interns who complete a cultural immersion opportunity** |
| - Overall reflection is incorporated throughout the program, not exclusive for an Indigenous community/setting placement. - Examples of reflection/debriefing techniques include talking circles, journal writing and medicine walks. |
| **PROFESSIONAL PRACTICE** |
| **Section D - Cultural safety and or anti-racism training for staff & faculty** |
| 1. **Type of cultural safety and/or anti-racism training provided by the program/institution provide to staff & faculty?** |
| - Mandatory workplace equity workshop for faculty - Cultural safety and anti-racism training provided through partner institutions (i.e., placement hosts) - External partnerships developed to provide anti-racism training (i.e., local municipalities) - Staff-maintained online forum to promote continuous learning (i.e., sharing events, resources) - Discussion groups and/or journal clubs - Movie/documentary nights. - Program and/or institutional resources to support staff and faculty (i.e., Indigenous Peoples webpage, guidelines for preceptors) |
| **Section E - Partnership with Indigenous Peoples (i.e., access to Elders/ Knowledge Keepers)** |
| 1. **Types of existing external partnerships with Indigenous Peoples communities and/or organizations in place in the program/institution** |
| - Can be led by a small number of Indigenous faculty and students, with the support of the program/institution’s administration. - Informal partnerships often exist through existent Indigenous faculty. - These partnerships can come with an additional role/committee for liaison purposes. |
| **STRATEGIC PLANNING, POLICIES, QUALITY IMPROVEMENT** |
| **Section F - Strategic planning** |
| 1. **Existence of a stratetic plan in the program/institution, focusing on Indigenization, decolonization and/or reconciliation** |
| - A strategic plan exists at the institutional level but there is lack of support for one at the program level. - Focused action plan to reduce barriers for Indigenous students |
| **Section G - Indigenous advisory board/council** |
| 1. **Existence of an Indigenous Advisory council, board, or group? In the program/institution** |
| - These groups can also exist within the organizations partnering with the program/ institution (i.e., placements’ hosts) - Often their role and work is done by internal Indigenous staff/departments, such as an Indigenous Initiatives Coordinator, and/or through existing partnerships with local Indigenous organizations. |
| **Section H - Policies regarding Indigenization, decolonization, and reconciliation** |
| 1. **Focus of the policies regarding Indigenization, decolonization, and reconciliation currently in place in the program/institution** |
| - Ensuring that the staff in charge of dealing with harassment and discrimination complaints are familiar with Indigenous Peoples’ experiences and needs. - Partnering with local Indigenous communities for ethics approval (for research projects). - Ensuring that the program/institutional ethics board includes Indigenous staff and/or faculty. |
| **Section I - Quality improvement (i.e., evaluation of current practices)** |
| 1. **Regular evaluation of action taken towards Indigenization, decolonization, and reconciliation in the program/institution** |
| - Evaluation takes place but as part of ongoing efforts, and for program accreditation purposes (not exclusive of Indigenous-focused action) |
| 1. **Actions that are subject to evaluation/monitoring in the program/institution** |
| - Success of Indigenous learning outcomes - Impact of exposing students to Indigenous leaders (supported by a teaching & learning grant) |
